# Clinical revenues of selective use of [18F]-FDG-PET/CT scanning in the management *of Staphylococcus aureus* bacteremia

**DOI:** 10.1007/s10096-025-05052-5

**Published:** 2025-02-08

**Authors:** Dewi Verkaik, Annette C. Westgeest, Jian Ling Wu, Kim C. E. Sigaloff, Merel M. C. Lambregts, Mark G. J. de Boer

**Affiliations:** 1 Leiden University Center of Infectious Diseases (LUCID), Leiden University Medical Center, Leiden, The Netherlands; 2 Department of Internal Medicine, Amsterdam UMC Location Vrije Universiteit Amsterdam, Amsterdam, The Netherlands; 3 Amsterdam Institute for Infection and Immunity, Amsterdam, The Netherlands; 4 Department of Clinical Epidemiology, Leiden University Medical Center, Leiden, The Netherlands; 5 Leiden University Center of Infectious Diseases, Leiden University Medical Center, Room C-5-44, Albinusdreef 2, Leiden, 2333 RZ The Netherlands

**Keywords:** *Staphylococcus aureus*, Bacteremia, [18F]-FDG-PET/CT, Sepsis

## Abstract

**Purpose:**

Although [18F]-FDG-PET/CT scanning (PET/CT) is recommended for managing *Staphylococcus aureus* bacteremia (SAB), its added value remains debated. This study investigated the clinical revenues of selective PET/CT use in SAB by considering three consequence-categories: detection of new infection foci, performance of new interventions, and alterations in antimicrobial therapy.

**Methods:**

All adult patients with ≥ 1 blood culture (BC) positive with *Staphylococcus aureus* admitted in a Dutch academic center between 2017^11^ and 2023^11^ were identified. Standard practice was to order PET/CT for patients with community acquired SAB and/or positive BCs after ≥ 48 h of treatment, or if multiple foci, or persistent fever, or endocarditis were present. Clinical- and laboratory data were obtained from electronic health records. Numbers-needed-to-scan (NNT-scan) were calculated for each consequence-category. Regression analyses were performed to identify variables correlated with consequence-bearing PET/CT.

**Results:**

Of 397 SAB patients, 143 (36%) underwent PET/CT. This led to detection of new foci in 73/143 patients (NNT-scan ≈ 2), new interventions in 33/143 patients (NNT-scan ≈ 4), and a change in antimicrobial therapy in 44/143 patients (NNT-scan ≈ 3). A CRP > 200 mg/L at presentation and positive follow-up BCs at 48 h were independently associated with interventions following PET/CT (adjusted OR and 95%CI 3.2 (1.2–8.3) and 2.6 (1.0-6.7) respectively). PET/CT results instigated changes in antimicrobial therapy predominantly in patients < 65 years and those with a CRP < 100 at presentation.

**Conclusions:**

Selective PET/CT ordering in real-life practice resulted in a relatively low NNT-scan across all consequence-categories. Further research is warranted to optimize patient selection for PET/CT using clinical parameters or profiles.

**Supplementary Information:**

The online version contains supplementary material available at 10.1007/s10096-025-05052-5.

## Introduction

*Staphylococcus aureus* bacteremia (SAB) is a severe bloodstream infection that is strongly associated with the presence of endocarditis and other metastatic infection foci. The 30-day mortality rate of SAB is approximately 20% and increases up to 35% after one year [1, 2]. In accordance with current guidelines for the management of SAB, this disease is usually classified as either uncomplicated or complicated. SAB is generally considered to be complicated if spondylodiscitis, endocarditis, or metastatic abscesses have been confirmed. In addition, several clinical characteristics are considered risk factors for complicated disease, such as a positive follow-up blood culture after at least 48 h of adequate treatment and/or the presence of prosthetic material [3, 4]. This classification is used to select an appropriate antimicrobial treatment dosage and duration [5].

Some current guidelines suggest or recommend the use of [18F] fluorodeoxyglucose positron emission tomography/computed tomography [18F]-FDG-PET/CT (PET/CT) scanning to aid in the classification and management of SAB [6, 7]. When a patient is either at risk for- or already diagnosed with - complicated disease, a PET/CT can be used to guide treatment strategy by newly detecting or confirming metastatic foci of infection [6]. Knowing the location and nature of infection foci is critical as they may require therapeutic interventions. A recent systematic review reported that the use of PET/CT is associated with a lower mortality rate and new diagnostic findings [8]. However, the studies included in this review had high degrees of bias and relatively low numbers of participants (*n* = 48) in the PET/CT group, which reduces the strength of the evidence presented [9]. Moreover, a recent post-hoc analysis of the effect of PET/CT on mortality revealed that the higher survival rates rates in patients who underwent PET/CT was mainly caused by immortal-time bias that was not accounted for [10].

Whether the use of PET/CT supports the management of patients with SAB most probably depends on the characteristics of the involved patient population, other varying factors such as infectious disease bedside consultation and whether other diagnostic modalities such as an echocardiogram, a CT of the thorax-abdomen or an MRI of the spine have been performed. A recent worldwide survey study identified large differences regarding the perceived indications for PET/CT use in patients with SAB [11]. The aim of this current study was to investigate the added clinical revenues of the use of PET/CT when scanning is performed selectively, i.e., in patients at the highest risk for metastatic foci.

## Methods

### Study design

This study was designed as a retrospective observational cohort study of SAB patients presenting at an academic tertiary care and referral hospital (Leiden University Medical Center, LUMC) in The Netherlands. To make an assessment on the added value of selective use of the PET/CT scan, three aspects were considered as outcome measures: detection of new infection foci, performance of new interventions, and alterations in dosage and/or duration of antimicrobial therapy. At the LUMC, PET/CT is normally ordered for patients with community acquired SAB and positive BCs after ≥ 48 h of adequate treatment; or if multiple foci, persistent fever, or endocarditis are present. To allow myocardial suppression analysis of the PET/CT images, a preparation protocol with 18 h of fasting plus two low carbohydrate meals preceding the 18 h fasting period is in place.

### Study population

All adult patients (age ≥ 18 years) with a blood culture (BC) positive for *S. aureus* between November 2017 and November 2023 were included. Patients were grouped based on whether they underwent PET/CT or not. Data was collected from the electronic health record database using the clinical data collection tool CTcue (CTcue, Iquvia B.V., Amsterdam, the Netherlands). CTcue is an AI-based program able to search the electronic health record database for specific combinations of terms, known as a query, using machine learning and natural language processing (NLP). All patients receive a pseudonym in this process, ensuring data privacy [12]. A query was developed to find all patients matching inclusion criteria, including repeated validation of search results. With the search results, CTcue also provides the relevant text fragment from the patient file, a so called ‘snippet’. Eligibility was confirmed by manual validation of this snippet of the blood culture report. When this text fragment did not provide sufficient information to confirm inclusion, this was validated by reviewing the electronic patient file around admission time.

### Study definitions

SAB was considered community acquired if a positive blood culture was obtained < 48 h after the time point of hospital admission. If the first positive blood culture was obtained > 48 h after admission, SAB was considered hospital acquired. Any intra- or extravascular devices or prosthetics, such as pacemakers, implantable cardiac defibrillators (ICD), prosthetic cardiac valves, prosthetic joints or fracture fixation materials were considered foreign material. The susceptibility of *S. aureus* strains was tested according to EUCAST criteria. Relapse of SAB was defined as a blood culture positive for *S. aureus*, obtained within 90 days after the last positive blood culture of the first bacteremia episode. Endocarditis was scored according to Duke’s criteria [13]. An infection focus was defined as any distinct anatomical site were *S. aureus* caused infection as first recognized either by clinical signs and symptoms, imaging modalities or both. Interventions were defined as any procedural medical action taken upon SAB related findings following PET/CT, e.g., abscess drainage, or conducting additional diagnostic procedures. Therapy alterations were defined as any change in the choice of antimicrobial agents, or the dosage or duration thereof. Besides the use of corticosteroid drugs, the use of other systemic immunosuppressive medication was taken into account as a co-variable and grouped as ‘immunosuppressors other than corticosteroids’. This determinant includes a broad range of immunosuppressive agents including - but not limited to - therapies directed at interfering in TNF pathways, interleukin blockade and complement inhibitors.

### Data collection

Baseline demographic and clinical parameters (within 48 h after the first positive blood culture) were collected from the EHR database. Patient eGFR was calculated, by taking the highest creatine measurement within 48 h of admission, using the CKD-EPI formula [14]. The highest value found for each clinical and laboratory parameter was used for final analysis. Subitems of each consequence category were manually scored by a team of two experienced clinician-scientists by reviewing patient files following a predefined procedure. Each researcher was instructed to independently review the reports from three days before up until seven days after PET/CT had been done, as well as reports of MRI, CT and echocardiography done within the respective admission period. Subsequently, (I) foci known before and newly discovered foci, and (II) diagnostic and therapeutic interventions, and (III) antimicrobial treatment alterations done following PET/CT were scored. Changes in antimicrobial treatment were considered to be instigated by PETC-CT results only if the medical file clearly indicated that the PET/CT result was the primary factor leading to the decision to initiate interventions or alter antimicrobial treatment. Cross-validation was performed in 10% of patients to account for interobserver variability.

### Statistical analyses

The primary outcome measure was the absolute number and proportion of patients in each of the consequence-categories following PET/CT. Data were presented as numbers, proportions and/or percentage. A number needed to scan (NNT-scan) was calculated for each of the outcome variables. For each of the outcome variables univariable tests (Mann–Whitney U test for continuous data, Chi-square test or Fischer’s exact test for categorical data) were used to assess which covariates show a significant association with the outcome variables. Parameters that showed an association and with a p-value of < 0.10 were included in the multivariable logistic regression analyses for each of the outcome variables. All statistical analyses were performed using IBM SPSS Statistics version 29.

### Ethical approval

Approval for this study was granted by the institutional ethics review committee of the Leiden University Medical Center, the Netherlands (protocol number 270923).

## Results

**Fig. 1:**
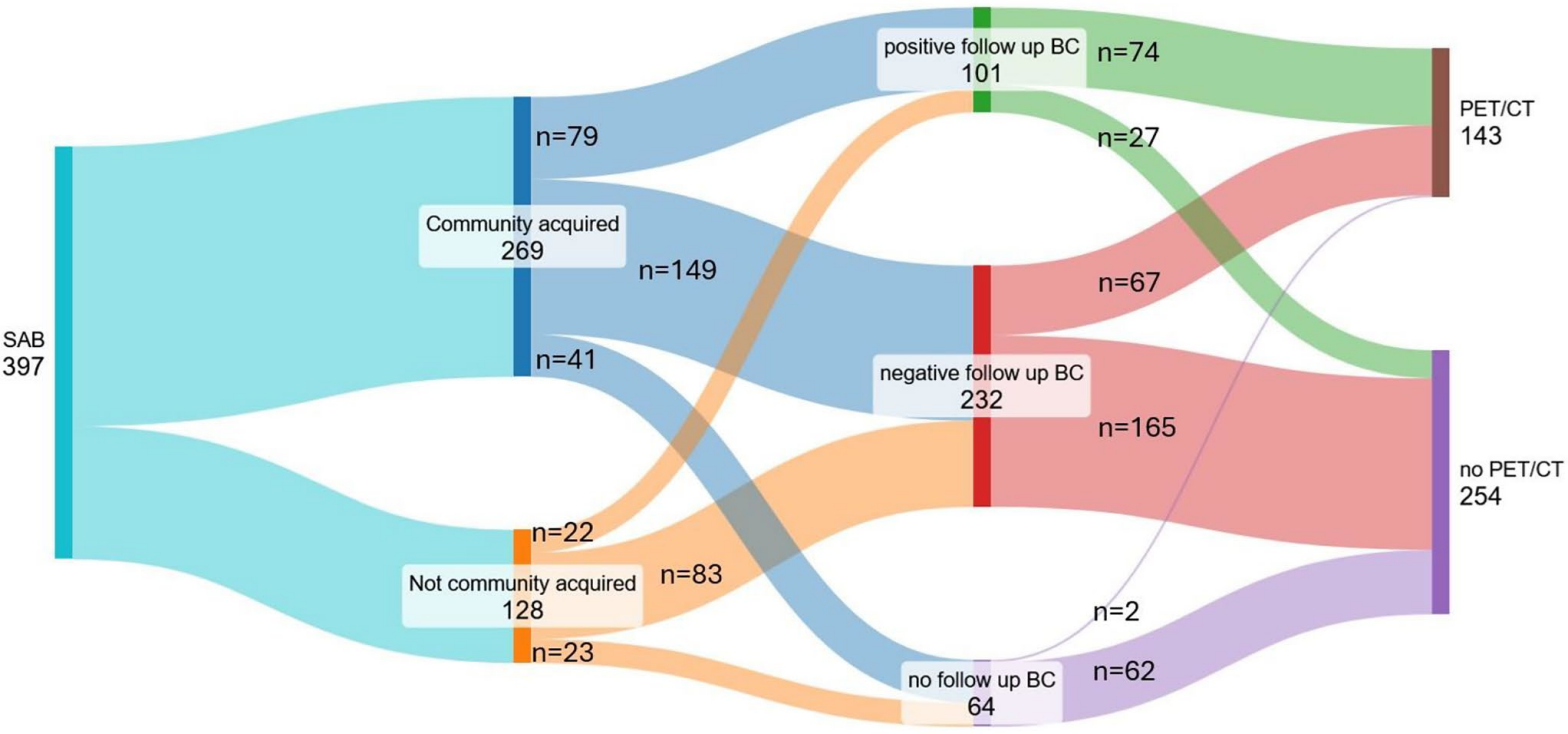
PET/CT performed in SAB patients according to origin and 48 h positive follow-up blood cultures. Legend: SAB: *Staphylococcus aureus* bacteremia; BC: blood cultures; follow up BC: Blood culture taken after approximately 48 h after the 1st positive BC and start of treatment

**Fig. 2:**
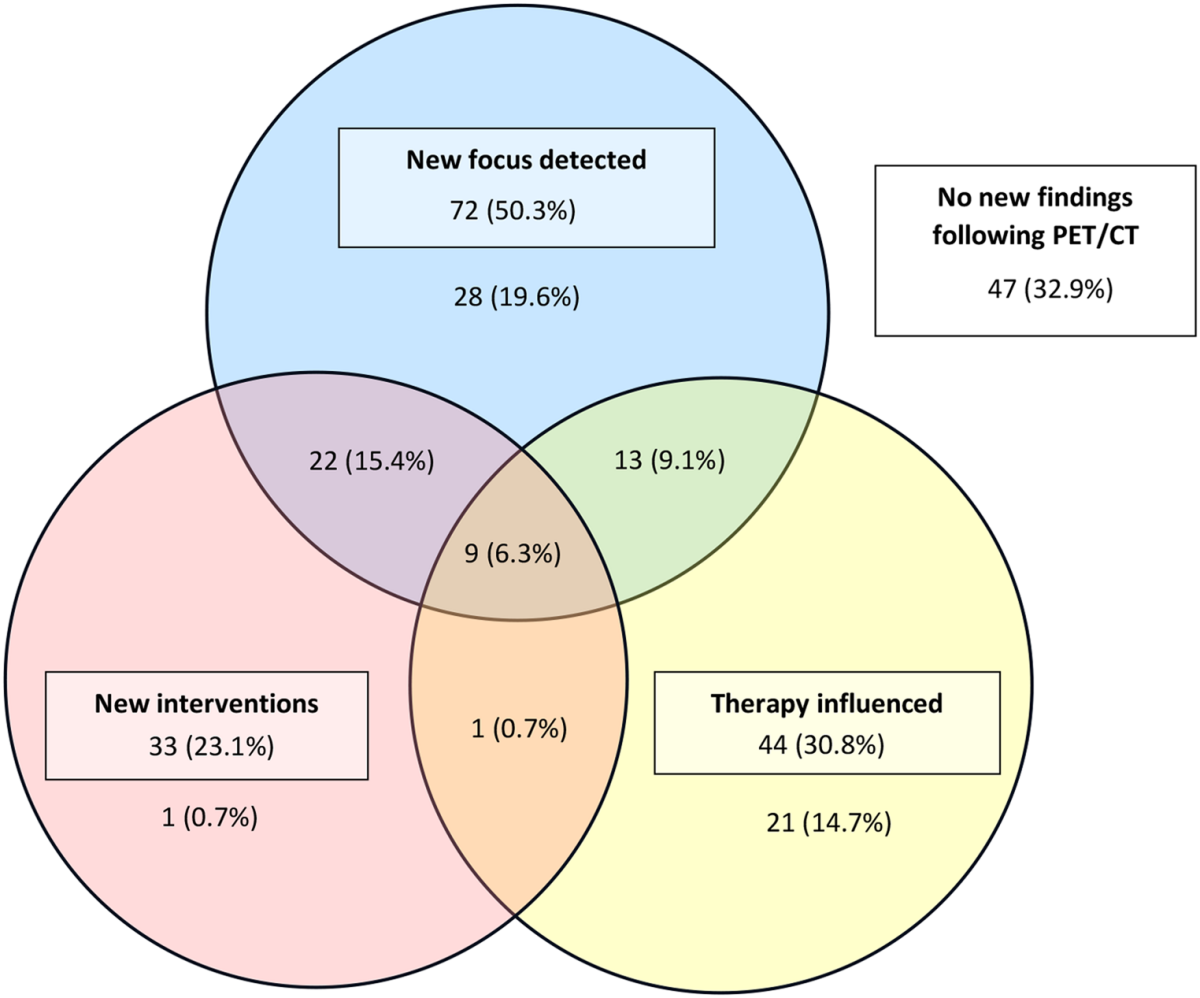
Venn diagram of the distribution of *S. aureus* bacteremia patients among the consequence categories

In total, 397 patients with SAB were included in this study (Fig. 1). The median age was 66 years, and 68% were male. Among all patients, there were four cases of a resistant *S. aureus* (MRSA or borderline oxacillin resistant *Staphylococcus aureus*, BORSA). Overall, the 90-day mortality was 27% (107/397) and the overall 90-day relapse rate was 1% (4/397). Out of all patients included in the study, 143 (36%) received a PET/CT (Fig. 2).

Demographic and clinical features of patients are shown in Table 1. In the group of patients in whom a PET/CT was performed, more comorbidities were present, such as dialysis dependence (15/143 vs. 13/254, *p* = 0.05), presence of foreign material (48/143 vs. 50/254, *p* = 0.002), and solid organ transplants (22/143 vs. 20/254, *p* = 0.02).

**Table 1:** Demographic and clinical features of included patients

	All patients	Patients who underwent [^18^F] FDG-PET/CT	Patients who did not underwent [^18^F] FDG-PET/CT	*p*-value
**Baseline characteristics**				
*Demographics*				
Total, n	397	143	254	-
Age, median (Q1– Q3)	66.0 (55.0–75.0)	67.0 (54.0–76.0)	66.0 (56.0–75.0)	0.83
Male, n (%)	269/397 (67.8)	101/143 (70.6)	168/254 (66.1)	0.36
BMI, median (Q1– Q3)	25.4 (23.1–28.8)	25.5 (23.0–28.5)	25.4 (23.1-29.01)	0.86
*Comorbidities*				
Diabetes mellitus, n (%)	125/397 (31.4)	55/143 (38.5)	70/254 (27.6)	0.03
Dialysis dependence, n (%)	28/397 (7.1)	15/143 (10.5)	13/254 (5.1)	0.05
Neutropenia, n (%)	20/397 (5.0)	8/143 (5.6)	12/254 (4.7)	0.70
Malignancy, n (%)	33/397 (8.3)	9/143 (6.3)	24/254 (9.4)	0.27
HIV, n (%)	1/397 (0.3)	0/143 (0.0)	1/254 (0.4)	1.00
Foreign material, n (%)	98/397 (24.7)	48/143 (33.6)	50/254 (19.7)	0.002
Organ transplant, n (%)	42/397 (10.6)	22/143 (15.4)	20/254 (7.9)	0.02
*Clinical parameters*				
MAP at presentation, median (Q1– Q3)	101 (90–112)	102 (93–115)	100 (89–111)	0.08
Heart rate, median (Q1– Q3)	95.0 (82.0–110.0)	95.0 (85.0–110.5)	96.5 (80.0-110.0)	0.66
Fever (> 38.5 C) in the first 48 h of admission, n (%)	245/359 (68.2)	94/136 (69.1)	151/223 (67.6)	0.78
Second positive blood culture after 48 h, n (%)	101/333 (30.3)	74/141 (52.5)	27/192 (14.1)	< 0.001
*Medication use*				
Corticosteroid, n (%)	140/297 (35.3)	49/143 (34.3)	91/254 (35.8)	0.76
Immunosuppressors (excluding corticosteroids), n (%)	42/397 (10.6)	18/143 (12.6)	24/254 (9.4)	0.33
*S. aureus susceptibility data*				
Flucloxacillin susceptibility*, n (%)	379/383 (99.0)	139/141 (98.6)	240/242 (99.2)	0.63
Clindamycin susceptibility, n (%)	343/397 (86.4)	121/143 (84.6)	222/254 (87.4)	0.44
*Laboratory inflammatory markers (maximum in 48 h window after drawing the first positive BC)*				
CRP, median (Q1– Q3)	178.4 (97.1–297.8)	202.1 (127.2–305.9)	158.8 (90.1–282.5)	0.01
Creatinine, median (Q1– Q3)	101.0 (71.0–182.5)	101.0 (72.8–190.0)	99.0 (71.0–172.5)	0.34
eGFR, median (Q1– Q3)	66.6 (33.6–95.2)	62.4 (28.6–94.9)	67.7 (34.3–95.8)	0.30
BUN, median (Q1– Q3)	10.4 (6.2–19.4)	11.3 (7.1–21.6)	9.7 (6.0–18.8)	0.12
Thrombocytes, median (Q1– Q3)	217.0 (156.5–297.3)	221.0 (152.5–296.8)	215.5 (158.8–298.3)	0.69
Leukocytes, median (Q1– Q3)	13.4 (9.5–17.9)	13.9 (10.8–17.7)	13.0 (8.9–18.0)	0.38
Neutrophils, median (Q1– Q3)	7.2 (2.9–10.3)	8.0 (5.4–12.7)	6.3 (2.6–9.8)	0.15
ESR, median (Q1– Q3)	59.5 (32.5–98.0)	68.0 (31.0–190.0)	50.0 (33.0–104.0)	0.65
*Route of acquisition*				
Community acquired, n (%)	269/397 (67.8)	121/143 (84.6)	148/254 (58.3)	< 0.001
**Follow-up data on diagnostics and hospital stay**				
*Diagnostic examinations*				
TTE, n (%)	225/397 (56.7)	125/143 (87.4)	100/254 (39.4)	< 0.001
TTE, showing endocarditis, n (%)	29/225 (12.8)	24/125 (19.2)	5/100 (5.0)	0.02
TEE, n (%)	58/397 (14.6)	41/143 (28.7)	17/254 (6.7)	< 0.001
TEE, showing endocarditis, n (%)	18/58 (31.0)	15/41 (36.6)	3/17 (17.6)	0.16
MRI spine, n (%)	44/397 (11.1)	27/143 (18.9)	17/254 (6.7)	< 0.001
CT abdomen-thorax, n (%)	111/397 (28.0)	44/154 (30.8)	67/254 (26.4)	0.35
*Admission data*				
Hospital stays (median, Q1– Q3)	16.1 (9.2–26.5)	19.6 (13.6–31.7)	14.1 (7.7–23.2)	< 0.001
Time between drawal of the 1st positive blood culture and [^18^F] FDG-PET/CT (days), median (Q1– Q3)	-	5.6 (4.3–8.3)	-	-
No. of positive blood cultures, median (Q1– Q3)	1.0 (1.0–3.0)	3.0 (1.0–4.0)	1.0 (1.0–2.0)	< 0.001
Total No. of blood cultures, median (Q1– Q3)	4.0 (3.0–7.0)	6.0 (4.0–9.0)	3.0 (2.0–5.0)	< 0.001
Days of positive blood cultures, median (Q1– Q3)	1.0 (1.0–1.8)	1.8 (1.0–3.1)	1.0 (1.0–1.0)	< 0.001
Intensive care admission, n (%)	119/397 (30.0)	35/143 (24.5)	84/254 (33.1)	0.07
ICU stay (days), median (Q1– Q3)	4.8 (1.0–12.8)	2.5 (0.8–9.8)	4.9 (1.2–13.0)	0.16

Legend

Susceptibility was tested according to EUCAST criteria; *MRSA or BORSA

Malignancy: Malignancy for which the patient was treated in the past 30 days

BMI: Body mass index, Fever: Body temperature > 38.5 °C, CRP: C-reactive protein, eGFR: Estimated glomerular filtration rate, Calculated according to CKD-EPI, BUN: Blood urea nitrogen, TTE: Transthoracic echocardiogram, TEE: Transesophageal echocardiogram, Q1: First quartile, Q3: Third quartile. Q1-Q3: Interquartile range

Most patients that underwent PET/CT had community acquisition of *S. aureus* (121/143, 84.6%). More blood cultures were taken (6 compared to 3, *p* < 0.001) in patients who underwent PET-CT, of which more were positive (3 compared to 1, *p* < 0.001). Patients imaged by PET/CT also had significantly more other diagnostic examinations done (TTE, TEE, MRI *p* < 0.001).

### Primary outcome

In 110 (76.9%) patients, there was at least one infection focus known before PET/CT. Among these foci, soft tissue infections (*n* = 50, 35.0%) and arthritis (*n* = 26, 18.2%) were most prevalent. PET/CT led to the discovery of one or more new/additional infection foci in 72 patients (50.3%). The overall NNT-scan to find any new focus of infection was approximately two (Table 2a). In total, PET/CT resulted in a new intervention in 33 patients (23.1%). Most of these interventions were a request of additional radiology exams, which often indicated to investigate potential drainage possibilities. The NNT-scan to perform an intervention was approximately four. When excluding the request of additional radiology exams as an intervention, the NNT-scan to perform an intervention was approximately 10. Interventions included abscess drainages (*n* = 9, 6.3%) and the removal of foreign material (*n* = 4, 2.8%). A change in antimicrobial therapy followed a PET/CT in 44 patients (30.8%). The duration of antimicrobial therapy was increased in 11 patients (7.7%) and decreased in 14 patients (9.8%). The dosage of antimicrobial therapy was increased in 16 patients (11.2%) and decreased in 21 patients (14.7%). The NNT-scan for a change in antimicrobial treatment strategy was approximately three (Table 2b). Consequence categories were scored with a concordance of 93% between researchers.

**Table 2a:** Numbers needed to scan by PET/CT (*N* = 143) to identify new foci of *S. Aureus* infection

	Foci identified before PET/CT, *n* (%)	New* foci identified by PET/CT, *n* (%)	Approximate NNT-scan (rounded numbers.)
**I Location of new foci**			
**Any focus**	**110 (76.9)**	**72 (50.3)**	**2**
Endocarditis	19 (13.3)	8 (5.6)	18
Spondylodiscitis	9 (6.3)	12 (8.4)	12
Osteomyelitis	13 (9.1)	15 (10.5)	10
Arthritis	26 (18.2)	14 (9.8)	10
Solid organ (excl. lung)	3 (2.1)	4 (2.8)	36
Lung	5 (3.5)	16 (11.2)	9
Soft tissue	50 (35.0)	38 (26.6)	4
Other ^	31 (21.7)	15 (10.5)	10

Legend: *: New foci: new site attributed to *S. aureus* infection, as identified by PET/CT and not known previously e.g., through clinical examination or other imaging modalities. NNT-scan: average number of patients needed to scan for detecting one new focus of infection. ^: Other foci of infection were (n before / n new): located in the central nervous system (2/2), vascular grafts (3/7), thrombophlebitis or infected thrombi (9/2), intravascular devices (6/2), central venous catheter related (7/0), not classifiable (4/2)

**Table 2b:** Numbers needed to scan by PET/CT (*N* = 143) to instigate new interventions

	Interventions following and instigated by PET/CT *n*, (%)	Approximate NNT-scan (rounded numbers)
**Interventions**		
**Any Intervention**	**33 (23.1)**	**4**
Any intervention (except additional radiology exams)	14 (9.8)	10
Abscess drainage	9 (6.3)	16
Additional radiology exams requested	30 (21.0)	5
Device removal	1 (0.7)	143
Line removal	1 (0.7)	143
Prosthetic material removal	2 (1.4)	72
Other surgical interventions	2 (1.4)	72
**III Change of antimicrobial treatment**		
**Any antimicrobial management change**	**44 (30.8)**	**3**
Antibiotic duration	25 (17.5)	6
Antibiotic duration increased	11 (7.7)	13
Antibiotic duration decreased	14 (9.8)	10
Antibiotic dosage	37 (25.9)	4
Antibiotic dosage increased	16 (11.2)	9
Antibiotic dosage decreased	21 (14.7)	7
Antibiotic switch	3 (2.1)	50

Legend: NNT-scan: average number of patients needed to scan leading to one intervention

### Factors associated with primary outcome measures

Univariable analysis showed an association between the detection of new infection foci on PET/CT and infection foci known prior to PET/CT, the use of corticosteroids, and a baseline eGFR < 30 (OR and 95%CI’s: 0.5 (0.2–1.1); 0.4 (0.2–0.9) and 2.4 (1.1–5.3) respectively). In the multivariable analysis, all showed an independent association with adjusted ORs of 0.4 (0.1–0.9), *p* = 0.03; 0.4 (0.2-1.0), *p* = 0.04; and 2.7 (1.1–6.5) *p* = 0.03 respectively.

Dialysis dependency (OR 4.7 95%CI 1.6–14.2), baseline CRP > 200 mg/L (OR 2.5 95%CI 1.1–5.6), and baseline blood urea nitrogen (BUN) > 7.5 mmol/L (OR 3.4 95%CI 1.1–10.5) were associated with new interventions instigated by PET/CT. Dialysis dependency and a baseline CRP > 200 mg/L showed an independent association in multivariable analysis, with an adjusted OR (95%CI)’s 6.0 (1.5–23.5), *p* = 0.01 and 3.2 (1.2–8.3), *p* = 0.02 respectively. A baseline CRP < 100 mg/L (OR 3.1 95%CI 1.3–7.4), age < 65 years (OR 2.2 95%CI 1.1–4.5), and positive 48 h follow up blood cultures (OR 0.3 95%CI 0.1–0.6) were associated with PET/CT influencing antimicrobial treatment. Multivariable analyses showed an independent association between positive 48 h follow up blood cultures and therapy alterations following PET/CT, with an aOR (95%CI) of 0.3 (0.3–0.6), *p* = 0.002 (Tables 3 and 4).

**Table 3:** Univariable analysis of factors associated with each consequence category

	Univariable analysis					
Variable	**New focus detected**		**Interventions** ^**#**^		**Antimicrobial Therapy alterations**	
	OR [95% CI]	p-value	OR [95% CI]	p-value	OR [95% CI]	p-value
Age < 65	1.1 [0.6–2.1]	0.79	1.1 [0.5–2.4]	0.78	2.2 [1.1–4.5]	*0.03*
Male gender	1.3 [0.6–2.8]	0.43	1.4 [0.6–3.4]	0.46	1.0 [0.5–2.2]	0.98
BMI > 30	2.1 [0.8–5.4]	0.11	1.6 [0.6–4.2]	0.30	0.8 [ 0.3–2.1]	0.64
*Comorbidities*						
Diabetes mellitus	0.9 [0.5–1.8]	0.81	1.1 [0.5–2.3]	0.90	0.8 [0.4–1.6]	0.47
Dialysis dependence	2.1 [0.7–6.6]	0.18	4.7 [1.6–14.2]	*0.003*	4.0 [ 1.3–12.0]	*0.01*
Neutropenia	1.0 [0.2–4.1]	1.00	1.1 [0.2–5.8]	1.00	4.1 [0.9–18.0]	*0.06*
Malignancy*	0.8 [0.2–3.0]	0.75	0.9 [0.2–4.8]	1.00	3.0 [0.8–11.9]	0.13
Organ transplant	1.0 [0.4–2.4]	1.00	1.7 [0.6–4.6]	0.29	1.1 [0.4–2.8]	0.91
Foreign material	1.4 [0.7–2.9]	0.32	1.0 [0.4–2.3]	0.97	0.8 [0.4–1.7]	0.50
*Diagnostic examinations*						
Fever > 48 h treatment start	1.3 [0.6–2.6]	0.53	1.1 [0.4–2.5]	0.91	0.9 [0.4–1.9]	0.78
Positive follow up blood culture	1.9 [1.0–3.8]	*0.05*	2.6 [1.1–5.9]	*0.02*	0.3 [0.1–0.6]	*< 0.001*
*Infection foci diagnosed prior to PET*						
Any	0.5 [0.2–1.1]	*0.08*	0.6 [0.3–1.5]	0.26	0.7 [0.3–1.6]	0.43
Endocarditis	0.9 [0.3–2.3]	0.78	0.6 [0.2–2.2]	0.56	-	-
Spondylodiscitis	2.1 [0.5–8.6]	0.31	0.9 [0.2–4.8]	1.00	0.3 [0.0–2.2]	0.28
Osteomyelitis	0.6 [0.2–1.9]	0.37	0.6 [0.1–2.8]	0.73	1.5 [0.4–4.7]	0.54
Arthritis	2.1 [0.9–5.2]	*0.09*	1.6 [0.6–4.2]	0.30	0.8 [0.3–2.1]	0.64
Solid organ	2.0 [0.2–22.6]	1.00	1.7 [0.1–19.2]	0.55	-	-
Lung	0.6 [0.1–4.0]	0.68	-	-	-	-
Soft tissue	1.0 [0.5–1.9]	0.95	0.8 [0.3–1.8]	0.52	1.1 [0.5–2.3]	0.82
Other	0.8 [0.3–1.7]	0.51	6.5 [2.1–20.0]	*< 0.001*	1.6 [0.5–4.7]	0.41
*Medication use*						
Corticosteroids	0.4 [0.2–0.9]	*0.02*	0.7 [0.3–1.5]	0.34	1.0 [0.5–2.1]	0.98
Immunosuppressors (excluding corticosteroids)	0.8 [0.3–2.1]	0.59	2.4 [0.9–6.9]	*0.09*	2.0 [0.7–5.4]	0.18
*Route of acquisition*						
Hospital acquired	2.0 [0.8–5.0]	0.15	1.0 [0.3–3.0]	0.97	2.2 [0.7–7.0]	0.17
*Laboratory inflammatory markers*						
CRP < 100	0.4 [0.2–1.0]	*0.04*	0.5 [0.2–1.6]	0.24	3.1 [1.3–7.4]	*0.01*
CRP > 200	1.7 [0.9–3.3]	0.13	2.5 [1.1–5.6]	*0.03*	0.6 [0.3–1.3]	0.20
eGFR < 30	2.4 [1.1–5.3]	*0.03*	2.0 [0.9–4.7]	0.10	1.2 [0.5–2.6]	0.73
BUN > 7.5	1.6 [0.8–3.5]	0.20	3.4 [1.1–10.5]	*0.03*	1.3 [0.6–3.0]	0.55
Thrombocytes > 450	1.4 [0.4–4.8]	0.55	1.1 [0.3–4.3]	0.90	0.4 [0.1–2.0]	0.26
Leukocytes > 10	1.7 [0.7–4.0]	0.21	1.2 [ 0.4–3.2]	0.77	0.4 [0.2–1.0]	*0.06*

Legend:

*: Malignancy for which was treated in the past 30 days. #: Any intervention, see Table 2b.Positive follow up blood culture: Blood culture taken after approximately 48 h after the 1st positive BC and start of treatment

BMI: body mass index, fever: body temperature > 38.5 °C, CRP: C-reactive protein, eGFR: estimated glomerular filtration rate, calculated according to CKD-EPI, BUN: blood urea nitrogen. CRP C-reactive protein stratified arbitrarily by cut-offs per 100 mg/dl

Underlined variables were included in multivariable analysis (*p* < 0.10)

**Table 4:** Multivariable analysis of factors associated with each consequence category

	Multivariable analysis					
**Variable**	**New focus detected**		**Interventions** ^**#**^		**Therapy alterations**	
	aOR [95% CI]	p-value	aOR [95% CI]	p-value	aOR [95% CI]	p-value
Age < 65	-	-	-	-	1.9 [0.8–4.3]	0.14
*Comorbidities*						
Dialysis dependence	-	-	6.0 [1.5–23.5]	0.01	3.4 [1.0–12.1]	0.06
Neutropenia	-	-	-	-	2.2 [0.4–11.6]	0.36
*Diagnostic examinations*						
Positive follow up blood culture	2.0 [0.9–4.2]	0.07	2.6 [1.0–6.7]	0.054	0.3 [0.1–0.6]	0.002
*Infection foci diagnosed prior to PET*						
Any	0.4 [0.1–0.9]	0.03	-	-	-	-
Arthritis	2.5 [0.9–6.8]	0.07	-	-	-	-
Other*	-	-	1.7 [0.6–4.8]	0.35	-	-
*Medication use*						
Corticosteroids	0.4 [0.2–1.0]	0.04	-	-	-	-
Immunosuppressors (excluding corticosteroids)	-	-	2.7 [0.8–9.4]	0.12	-	-
*Laboratory inflammatory markers*						
CRP < 100	0.4 [0.2–1.1]	0.09	-	-	2.4 [0.9–6.6]	0.08
CRP > 200	-	-	3.2 [1.2–8.3]	0.02	-	-
eGFR < 30	2.7 [1.1–6.5]	0.03	-	-	-	-
BUN > 7.5	-	-	1.9 [0.6–6.5]	0.28	-	-
Leukocytes > 10	-	-	-	-	0.7 [0.2–1.9]	0.48

Legend: #: Any intervention, see Table 2b. Positive follow up blood culture: Blood culture taken after approximately 48 h after the 1st positive BC and start of treatment; BMI: body mass index, CRP: CRP C-reactive protein stratified arbitrarily by cut-offs per 100 mg/dl., eGFR: estimated glomerular filtration rate, calculated according to CKD-EPI, BUN: blood urea nitrogen. *: Other meaning: none of the foci specifically listed in Table 3

## Discussion

We found that in approximately two thirds of selected patients who underwent a PET/CT, the radio-diagnostic procedure resulted in one or more consequences for their management. New foci of infection were discovered in 51% of patients, and these new findings led to a new intervention and/or increase of antimicrobial treatment dosage and/or duration in 30.8% of patients. The absence of new diagnostic findings on the PET/CT regularly led to alterations in antimicrobial treatment dosage and duration as well, i.e., mostly de-escalations. This can be explained by the observation that in clinical practice, PET/CT was often used in patients who had one or several risk factors for complicated disease, but no apparent clinical manifestations of endocarditis or metastatic infection foci. If no new foci were detected by PET/CT, physicians may have felt assured that a shorter duration of therapy was safe. This also shows that, while PET-CT results can be a key driver of the medical team’s decisions to perform an intervention or change therapy, other variables, such as the patient’s clinical condition, play a relevant role as well.

The detection of a new infection focus did not lead to any traceable intervention or therapy alteration in 20.3% of patients. Although there may not be a direct objectifiable consequence of detection of a new metastatic focus of infection, its discovery may still have contributed to the clinicians’ understanding of the disease in the individual patient and, thereby, to the overall care that was provided. This is a variable that is difficult to scientifically quantify.

Results from a previous study already suggested that treatment duration can be safely de-escalated in ‘high-risk’ SAB patients without signs of metastatic infection on PET/CT and absence of signs of endocarditis on echocardiography [15]. Hence, the use of PET/CT to guide the medical management in SAB patients with risk factors for complicated disease, but in whom complicated disease has not yet been proven nor excluded, may offer a niche for the application of PET/CT.

In another study [16], PET/CT was recommended for patients with risk factors for complicated disease based on similar criteria as in our study. PET/CT was performed in 66.9% of patients with risk factors for complicated disease. Here, the authors reported treatment modifications in 74.6% of patients. Alterations in therapy dosage and/or duration as well as surgical or radiological interventions were considered as treatment modifications. Surgical or radiological interventions ensued in 19.2% of patients, compared to 9.8% in the current study. Despite that similar risk factor criteria were used, fewer surgical or radiological interventions ensued in the current study for which - apart from case-mix heterogeneity - we could find no clear explanation.

While there are guidelines that offer recommendations for the use of PET/CT, these are not strongly supported by scientific evidence. Due to the complex nature of SAB, other factors can influence the decision to perform a PET/CT, such as overall clinical presentation or the clinical course after admission. For example, from our data it was derived that PET/CT was performed less frequently in ICU patients with SAB (data not shown), which can be explained by challenges associated with patient mobility due to medical equipment and compromised health conditions.

Previous studies reported that new infection foci were found by PET/CT in 70.8% and 45.8% of patients [9, 16]. In both studies, bone- and joint infections (38.4% − 33.3%) comprised the largest groups of newly detected foci. In the current study, although osteomyelitis and arthritis were also found often, PET/CT mostly led to the discovery of soft tissue and lung foci (Table 2a). Another study from the Netherlands, also most frequently found tissue and lung foci [17]. This shows that the frequency and/or type of newly discovered infectionfoci is dependent on indications used for PET/CT use, as well as diagnostic work-up prior to PET/CT, but possibly also on geographical location influencing the case-mix.

To optimize the utilization of PET/CT in the context of personalized diagnostic assessment of patients with SAB, associations between baseline clinical factors and clinically relevant outcomes of PET/CT need to be considered. Preceding studies found a positive association between CRP and the detection of new foci, which this current study was able to replicate [16]. In this study, high CRP levels led to more new discoveries, while low CRP levels were protective for the detection of new foci. Notably, in our study, an association was found between the use of corticosteroids at presentation and fewer new infection foci discovered by PET/CT. This could be partly due to the anti-inflammatory properties of corticosteroids that could have complicated the detection of infectionfoci by PET/CT.

Regarding the implementation of PET-CT for patients with SAB, some previous studies have showed an association between performing PET/CT and a lower mortality rate [9, 17]. However, this difference in mortality can be attributed to immortal time bias because patients who passed away in the first week after SAB acquisition probably did not yet underwent PET/CT scanning [10]. When assessing mortality after the first week in the current study, no considerable difference in mortality rate was observed (Supplementary Figs. 1, 2).

### Strengths and limitations

The main strength of this research is its novel and extensive approach to investigating the revenues of PET/CT for SAB patients by considering three different clinically relevant aspects. Clinical data and revenues of PET/CT were evaluated by two independent expert clinicians and cross-checked, but subjectivity in determining what was a focus of infection or not, can never be fully excluded. The use of an AI based clinical data collection tool allowed for rapid, reliable, and systematic collection of patient data. Multiple steps of thorough data validation have been implemented to minimize the amount of missing data and capture errors, resulting in a complete and comprehensive database. The results of this study were obtained in a single academic tertiary care center and therefore may not be generalizable to other settings with different patient populations. Another potential limitation is the selection introduced by the real-life practice setting of the study, were not all patients with indications for PET/CT scans ultimately received a scan for various reasons e.g., non-adherence to ID-consultation recommendations or death, and not in all patients new BC cultures were obtained 48 h after start of treatment. This could lead to an overestimation of the information obtained- and actions instigated by PET/CT, as the selected patients may not represent the entire population of eligible SAB patients according to the local protocol or guideline. On the other hand, the design may also enhance the generalizability of the results, as findings were based on a real-life clinical practice setting. The reported 90-day relapse rate (1%) may be underestimated because patients with a relapse may incidentally have presented in a different hospital, without notification.

One of the aims of this study was to understand which clinical variables are associated with a consequential outcome of PET/CT. Despite this study being limited to showing an association, and not causation, the identified associations could still be applicable in a clinical setting. To draw causal conclusions, future research is warranted in the form of a diagnostic randomized controlled trial (RCT), with a group of patients being randomly divided between getting a PET/CT or not. This would better allow patient-specific predictions on PET/CT benefit and increase specificity of guidelines for PET/CT use. Furthermore, by performing a RCT, mortality and relapse rates can be more fairly assessed, as well as quality of life and cost-efficiency.

### Summary and conclusions

PET/CT led to the identification of new infection foci (NNT-scan = 2), the performance of new interventions (NNT-scan = 4), and alterations in therapeutic dosage and/or duration (NNT-scan = 3) in a substantial number of high-risk patients. The NNT-scan, although estimates from a single center study, were clinically acceptable, as relatively few patients had to undergo PET/CT to result in a new positive finding in one or more of these categories. Dialysis dependency and high CRP levels at baseline showed a strong association with interventions following PET/CT. To confirm these findings and to increase predictability of actionable PET/CT outcomes, a prospective diagnostic trial is needed.

## Electronic supplementary material

Below is the link to the electronic supplementary material.

Supplementary Material 1

## Data Availability

No datasets were generated or analysed during the current study.
