# Supplementary material for: Clinical revenues of selective use of [18F]-FDG-PET/CT scanning in the management of Staphylococcus aureus bacteremia
Source: Eur J Clin Microbiol Infect Dis. 2025 Feb 8;44(4):895–904. doi: 10.1007/s10096-025-05052-5 (PMC11946999; doi:10.1007/s10096-025-05052-5)
Supplement: Supplementary file 1 — Supplementary Material 1 [file 10096_2025_5052_MOESM1_ESM.docx]

Supplementary file I.


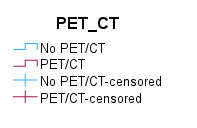

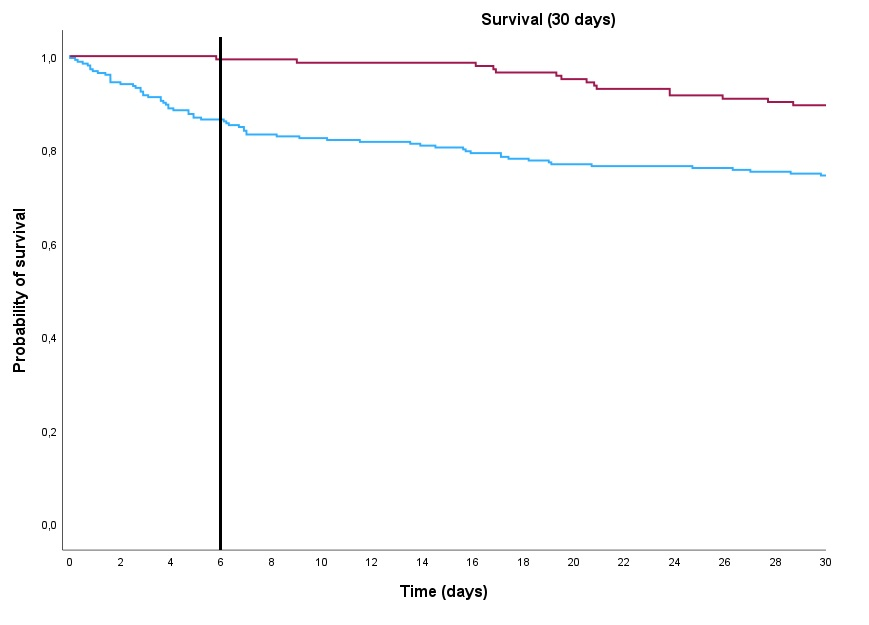
**Supplementary figure 1. Survival in *S. aureus* bacteremia patients that did or did not receive a PET/CT.**

Kaplan-Meier curve representing survival of patients with *S. aureus* bacteremia at 30 days. The black line represents day six, which was the median time to PET/CT (Q1 4.3 – Q3 8.3).

**Supplementary figure 2. Survival in *S. aureus* bacteremia patients that did or did not receive a PET/CT (adjusted for immortal time bias).**


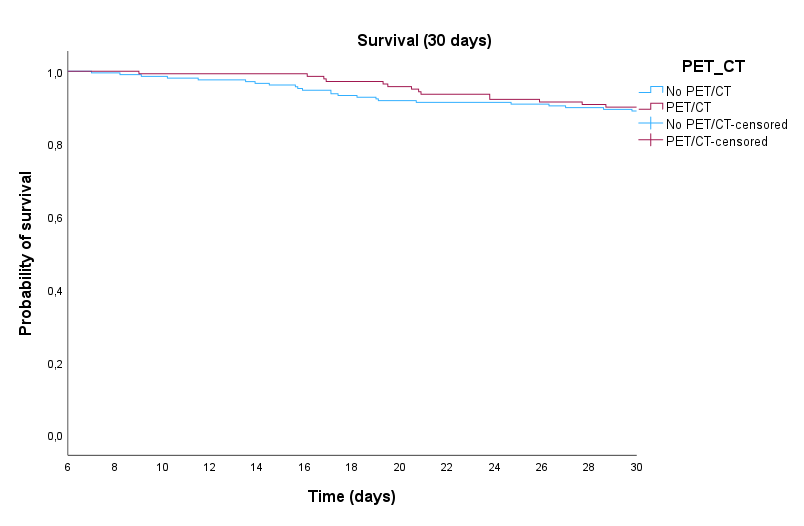


Kaplan-Meier curve representing survival of patients with *S. aureus* bacteremia at 30 days, without patients that passed away in the first six days, which was the median time to PET/CT (Q1 4.3 – Q3 8.3).
